# Letter to the Editor

**DOI:** 10.52054/FVVO.16.3.040

**Published:** 2024-09-30

**Authors:** PR Koninckx, A Ussia, B Amro, A Wattiez, L Adamyan

**Affiliations:** Prof emeritus Obstetrics and Gynecology KULeuven, Leuven, Belgium, the University of Oxford, Oxford, UK, Università Cattolica, Rome, Italy and Moscow State University, Moscow, Russia; Gruppo Italo Belga, Villa del Rosario, Rome Italy; Larifa Hospital, Dubai, UAE; University of Strasbourg, France; Department of Operative Gynecology, Federal State Budget Institution V. I. Kulakov Research Centre for Obstetrics, Gynecology, and Perinatology, Ministry of Health of the Russian Federation, and Department of Reproductive Medicine and Surgery, Moscow State University of Medicine and Dentistry, Moscow, Russia

Dear Editor,

The article ‘Non-invasive imaging techniques for diagnosis of pelvic deep endometriosis and endometriosis classification systems: an International Consensus Statement’ is an excellent compilation of reported sensitivities, specificities and likelihood ratios of the value of imaging for the diagnosis of deep endometriosis (Condous et al., 2024). Also, the consensus statements reflect clinical experience. However, some concerns should be addressed.

The reader should realise the overall poor methodological quality of these studies, as identified before (Nisenblat et al., 2016). First, the results are conditional on a laparoscopy being performed. Since only women undergoing a laparoscopy can be included, more women with severe pain and fewer women without symptoms will be included. Second, in the absence of blinding the surgeon to imaging results, imaging could have contributed to the indication to perform a laparoscopy. For both reasons, false negatives and negative predictive values are unknown, and reported sensitivities and specificities risk being (slightly) overestimated.

Another problem is that the prevalence of deep endometriosis was not considered. The word prevalence was not used in the presentation at Gynitaly24 and does not occur in the article. Sensitivities, specificities and likelihood ratios are independent of prevalences, but predictive values of a test vary with prevalences (Koninckx et al., 2021; Koninckx et al., 2023) and decrease sharply for prevalences less than 10%. This is illustrated by ‘if you hear hoofbeats, think of horses, not zebras’. Using conditional probabilities or Bayesian statistics, the positive predictive value (PPV) equals (sensitivity*prevalence)/ ((sensitivity*prevalence) + (1-specificity)*(1-prevalence)) (Lesaffre et al., 2012). Therefore, it should be explained which prevalences were used to calculate PPVs since prevalences of deep endometriosis vary from 2-4% in the population to >10% in referral centres because of referrals or even higher in women with (very) severe pain scheduled for surgery. The reported sensitivities and specificities of 85% and 94% (Condous et al., 2024) would result in PPVs of 30% for prevalences of 3% but over 90% for prevalences higher than 50%. The authors should discuss the inclusion criteria used in these studies. The results might be valid only for symptomatic women with (severe) pain, eventually a positive clinical exam, and already planned for surgery. Finally, when discussing the environmental aspect, it would be nice to consider the added value of MRI after a transvaginal ultrasound examination or vice versa (Koninckx et al., 2024).

The consensus statements reflect clinical experience and authority. The authors vary from surgeons to ultrasonographers to radiologists. As suggested (Koninckx et al., 2013), consensus opinions should be corrected for experience. Otherwise, authority-based “conclusions have the value of an educated opinion of the participants, without reflecting the opinions of those not participating”. Those without experience should be excluded, and experience should reflect collective experience, i.e. corrected for the individual experience (Wattiez et al., 2023). As a second problem, we wonder how consensus opinions can be formulated as levels or grades of evidence.

In conclusion, without discussing the clinical value of imaging for deep endometriosis surgery, methodological problems should be realised. We should know whether imaging results were used for the indication of surgery and whether the surgeon used imaging results when deciding to do surgery. Equally important is which prevalences should be used to estimate predictive values, how experience is defined and implemented for consensus opinions, and how (collective) experience-based opinions can be translated into grades of evidence. For these reasons, peer-reviewing of manuscripts remains important, however esteemed the authors and societies might be.
